# Evaluation of Internet users’ interest in paediatric dental problems during the COVID-19 pandemic

**DOI:** 10.1186/s12903-023-02815-4

**Published:** 2023-02-17

**Authors:** Emre Aksoy, Emine Sen Tunc

**Affiliations:** 1 Independent Researcher, Samsun, Turkey; 2 Department of Paediatric Dentistry, Faculty of Dentistry, Ondokuz Mayis University, Samsun, Turkey

**Keywords:** COVID-19, Google Trends, Paediatric dentistry

## Abstract

**Background:**

The coronavirus (COVID-19) pandemic had a significant impact on dental care providers; patients had trouble accessing routine dental care due to concerns about dentist and patient safety. People spent more time at home due to lockdown restrictions and a growth in individuals working from home. This increased the likelihood of looking for dental care information on the Internet. In the present study the aim was to compare the trends of Internet searches about paediatric dentistry before and after the pandemic.

**Methods:**

The monthly variation in relative search volume (RSV) and the lists of paediatric dentistry-related queries were determined from December 2016 to December 2021 using Google Trends. Two separate datasets were obtained before and after the pandemic. One-way ANOVA was used to determine whether there was a significant difference in RSV scores between the first 2 years of COVID-19 and the first 3 years before COVID-19. T-tests were used for bivariate comparisons.

**Results:**

There was a statistically significant increase in queries regarding dental emergencies, especially toothache (*p* < 0.01) and dental trauma (*p* < 0.05). The RSV of paediatric dentistry queries increased over time (*p* < 0.05). Other queries about recommended dental procedures during the pandemic, such as the Hall technique and stainless steel crowns, showed an increasing trend. However, these were not statistically significant (*p* > 0.05).

**Conclusions:**

More searches were carried out, related to dental emergencies, on the Internet during the pandemic. Moreover, non-aerosol generating procedures such as the Hall technique became increasingly popular according to the frequency of searches carried out.

## Background

COVID-19 is a novel coronavirus disease that emerged for the first time in the Chinese city of Wuhan. The World Health Organization (WHO) declared a pandemic on March 11, 2020, as a consequence of COVID-19’s rapid spread across many countries. COVID-19 has a high potential for spreading and is transmitted through small droplets [1]. The pandemic affected many health care systems, such as dental care. Many countries, including the United States and the United Kingdom, opted to implement lockdowns to avoid an overburdened healthcare system. In addition, due to concerns about the safety of dentists and patients, routine dental care had to be restricted [2].

The WHO stated that oral diseases are a serious public health problem, and the most common noted oral diseases are dental decay, tooth loss, periodontal disease, and trauma. Tooth caries affects 60–90% of school-aged children worldwide. In addition, since the early 1970s, the prevalence of caries in preschool-aged children has increased due to the higher consumption of sugar-containing diets [3, 4]. Dental trauma is a common oral problem that affects children and can lead to tooth loss [5]. In recent years, the Internet has provided a wealth of information. Individuals are able to access data quickly and efficiently through Internet search terms [6]. Internet platforms contain extensive information about self-medication, alternative treatments, and urgent dental care, and people with dental problems may search for information on the Internet to manage dental pain [7]. Google is the most popular search engine among Internet users. As a result, people are turning to Google as their primary source of dental information [8].

The COVID-19 pandemic had a significant impact on dental care providers; patients had difficulty accessing routine dental care due to concerns about the safety of both dentists and patients. People spent more time at home due to lockdown restrictions and many started working from home. This increased their likelihood of looking for dental care information on the Internet [2, 9]. Google Trends (GT) is a Google product that analyzes the popularity of Internet search terms and can contribute to the evaluation of dental problems in communities across different languages and regions worldwide [10]. GT can show what people's dental needs were during the pandemic, by comparing the first 2 years of COVID-19 with the first 3 years before COVID-19. In view of the lack of research on this subject, the aim in the present study was to compare the trends of Internet searches for paediatric dentistry queries before and after COVID-19.

## Methods

In this cross-sectional study, GT was used to evaluate Internet search trends during the COVID-19 pandemic worldwide. GT was used to collect data. When a person searches for a term using the Google search engine, GT generates data on how common the query term is. It presents normalized data about anonymous user searches in the form of relative search volumes (RSVs), varying from 0 to 100, where 100 represents the maximum value of searches in a given period.

The comparison strategy was based on standard terms among oral health policies and recommendations regarding paediatric dentistry defined by the American Academy of Pediatric Dentistry (AAPD) and related popular search queries as posted on GT [11]. Efforts were made to include terms that are likely to be familiar to people looking for information on dental care. Individuals may not utilise certain dental terminology such as regenerative pulp therapy. Therefore terminology such as, ‘space maintainer’ was included instead of ‘band and loop space maintainer’, and its RSV was higher. The paediatric dentistry-related queries are presented in Table 1. Each term was individually searched using GT. The monthly RSVs and the lists of paediatric dentistry-related queries were included from December 2016 to December 2021 using GT. The GT search parameters used were “Worldwide,” “December 2016–December 2021,” “All categories,” and “Web Search.” The datasets were analyzed using SPSS version 25 (IBM Corp, Armonk, NY, USA). The Shapiro–Wilk test was used to confirm the normality of distribution for each dataset per query. Two separate datasets were obtained, before and after the pandemic. One-way ANOVA was used to determine whether there was a significant difference in RSV scores between the first 2 years of COVID-19 and the first 3 years before COVID-19 worldwide. T-tests were used for bivariate comparisons. For all analyses, *p* < 0.05 was considered statistically significant.

**Table 1:** Top relative search volumes of paediatric dentistry related queries

Paediatric dentistry related diseases and treatments	Search terms with top RSV's
Early childhood caries	Baby teeth caries, child teeth caries, baby tooth caries, child tooth caries
Kids toothache	Kids toothache, child toothache
Dental trauma	Avulsed tooth, fractured tooth, broken tooth, broken teeth
Cleft lip and palate	Cleft lip, cleft palate, harelip
Root canal on baby teeth	Paediatric pulpotomy, baby root canal, root canal on baby tooth
Paediatric restorative dentistry	Hall technique, stainless steel crown, fluoride treatment, atraumatic restorative treatment, kids tooth filling
Space maintainer	Band and loop space maintainer, distal shoe space maintainer, transpalatal arch, lingual holding arch
Mouthguard	Sports mouthguard, kid mouthguard, child mouthguard
Paediatric dentistry	Pedodontist, child dentist, child dentistry, paediatric dentist

## Results

Many paediatric dentistry-related queries showed an increasing trend during the pandemic.

**Fig. 1:**
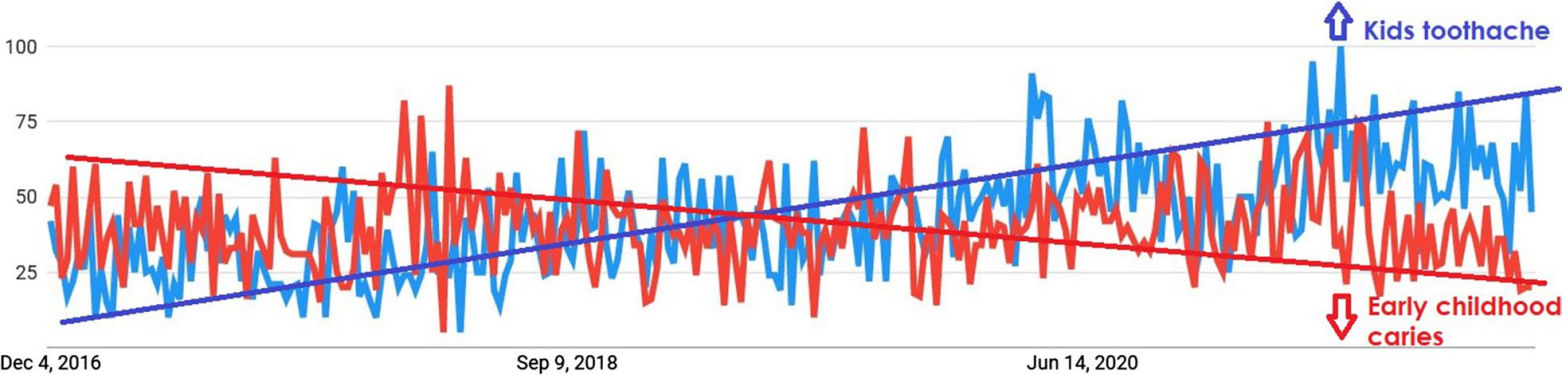
Comparative RSVs of kids toothache and early childhood caries

There was a decrease in the RSVs for three search queries, about early childhood caries, root canals on baby teeth, and mouthguards (Table 2). As shown in Fig. 1, although there was a noticeable increase in the RSV of kids’ toothache (*p* < 0.001), there was a decrease in the RSV of early childhood caries.

**Table 2:** Compared queries before the COVID-19 with during the COVID-19,

	Before the COVID-19	During the COVID-19	
	N = 36 month, M (SD)	N = 24 month, M (SD)	*t* (*df*)
Early childhood caries	25.81 (3.77)	25.08 (2.50)	0.824 (58)
Kids toothache	44.81 (11.36)	73.38 (11.29)	− 9.564 (58)**
Dental trauma	69.11 (8.13)	75.38 (4.93)	− 3.376 (58)**
Cleft lip and palate	24.47 (1.34)	29.50 (16.26)	− 1.854 (58)
Root canal on baby teeth	59.97 (10.12)	54.08 (11.70)	2.074 (58)
Hall technique	20.67 (7.54)	24.54 (9.87)	− 1.722 (58)
Stainless steel crown	39.75 (5.49)	41.75 (6.31)	− 1.301 (58)
Space maintainer	70.19 (9.94)	78.42 (14.68)	− 2.590 (58)*
Mouthguard	53.08 (9.24)	43.21 (10.08)	3.910 (58)**
Pediatric dentistry	75.81 (11.40)	81.42 (14.39)	− 1.680 (51)

*M* mean, *SD* standard deviation

*Indicates that the statistical significance level is *p* < .001, **indicates that the statistical significance level is *p* < .005

There was a statistically significant increase in the queries about dental emergencies, especially toothache (*p* < 0.01) and dental trauma (*p* < 0.05). The RSV of paediatric dentistry queries increased over time (*p* < 0.05). In addition, other queries about recommended dental procedures during the pandemic, such as the Hall technique and stainless steel crowns, showed an increasing trend. However, these were not statistically significant (*p* > 0.05). The results of the one-way ANOVA are presented in Table 3.

**Table 3:** Compared queries for the first 2 years of the COVID-19 with the first 3 years before the COVID-19

	Before the COVID-19, M (SD)			During the COVID-19, M (SD)		
	3 year before	2 year before	1 year before	1st year	2nd year	F
Early childhood caries	21.75 ± 1.76^d^	25.75 ± 0.97^b^	29.92 ± 2,19^a^	23.17 ± 1.76^c^	27.00 ± 1.48^b^	44.542**
Kids toothache	46.25 ± 8.80^b^	46.75 ± 13.03^b^	41.42 ± 12.03^b^	73.42 ± 7.24^a^	73.33 ± 14.64^a^	22.702**
Dental trauma	70.58 ± 5.25^ab^	65.16 ± 6.04^b^	71.58 ± 10.97^a^	74.50 ± 5.42^a^	76.25 ± 4.45^a^	4.65*
Cleft lip and palate	24.00 ± 1.35^b^	24.50 ± 1.24^b^	24.92 ± 1.38^b^	23.67 ± 2.31^b^	35.33 ± 21.75^a^	3.065
Root canal on baby teeth	58.00 ± 9.64^a^	61.25 ± 12.37^a^	60.67 ± 8.56^a^	51.75 ± 9.94^a^	56.42 ± 13.24^a^	1.479
Hall technique	20.33 ± 6.53^a^	22.33 ± 7.44^a^	19.33 ± 8.81^a^	25.33 ± 8.38^a^	23.75 ± 11.50^a^	0.95
Stainless steel crown	37.91 ± 6.49^b^	41.33 ± 4.29^ab^	40.00 ± 5.39^ab^	39.83 ± 5.69^ab^	43.67 ± 6.56^a^	1.644
Space maintainer	63.42 ± 5.66^b^	67.67 ± 6.46^b^	79.50 ± 9.43^a^	70.75 ± 17.09^b^	86.08 ± 5.53^a^	10.432**
Mouthguard	56.83 ± 11.30^a^	50.75 ± 7.15^ab^	51.67 ± 8.34^ab^	41.33 ± 10.59^bc^	45.08 ± 9.62^c^	4.819*
Pediatric dentistry	69.42 ± 8.80^c^	74.58 ± 10.54^bc^	83.42 ± 10.77^ab^	75.67 ± 16.60^bc^	87.17 ± 9.22^a^	4.604*

*M* mean, *SD* standard deviation

^a,b,c^The values represented by different letters on the same line are statistically different

*Indicates that the statistical significance level is *p* < .001; **indicates that the statistical significance level is *p* < .005

## Discussion

This is the first study in which GT queries were used to compare the trends of Internet searches about paediatric dentistry during the COVID-19 pandemic. In addition, there is limited knowledge about paediatric dentistry queries [12, 13]. Recent studies during the pandemic indicate greater interest in self-treatment for dental problems around the world [14, 15]. Therefore, the study is important as it show trends in Internet searches about paediatric dentistry during the pandemic.

COVID-19 has had an unprecedented global impact. Countries were obliged to implement various measures to control COVID-19, including restrictions on the health and dental sectors. Dental authorities around the world, such as the American Dental Association (ADA), AAPD, and British Dental Association, recommended postponing elective and non-urgent dental procedures at the beginning of the pandemic [16]. Dental care providers accepted only patients requiring urgent dental treatments and procedures [17]. Pericoronitis, oral trauma, severe toothache, acute abscesses, and life-threatening tissue bleeding were defined as oral emergencies [18]. Patients had difficulty accessing dental care during this time. Due to the unpredictable duration of the pandemic, new approaches and management procedures have become mandatory for routine dentistry practices. Paediatric dental practice in the post-COVID era is beginning to be routine [15, 19, 20]. Although people have access to dental care, the current situation regarding the COVID-19 pandemic has created panic in people's minds about whether to go to the dentist or stay at home and seek dental care on the Internet until the world returns to normal.

Various factors can cause toothaches, both odontogenic and non-odontogenic, but odontogenic factors are the more common cause. Toothache is a sign of poor oral health and it can negatively impact one's quality of life [21]. Lotto et al.'s study shows a continuous increase in the interest of Internet users in toothache queries over the years. Our results are in line with that study [22]. Moreover, we found a statistically significant increase during the pandemic. Another clinical condition that affects children and is linked to toothache is dental trauma. Dental trauma is a significant health issue among children, and it has the potential to degrade their quality of life [23]. In the present study, dental trauma was the term searched for most overall during the pandemic. The consequences of dental trauma can be serious, resulting in an irreversible dental loss at the time of the incident, during treatment, or even years later. Thus, new healthcare approaches for early diagnoses, such as teledentistry, are critical when access to dental care providers is limited, such as during pandemics.

Non-aerosol generating procedures for dental management of paediatric patients are recommended in the post-COVID era. These include atraumatic restorative treatment (ART), fissure sealants, silver diamine fluoride (SDF), the Hall technique, and interim therapeutic restorations. ART is a technique that involves removing caries selectively with hand instruments and filling them with a high-viscosity glass ionomer cement [24]. It is considered an important approach for treating children in the post-pandemic era [16]. The Hall technique is a non-invasive method of restoring carious primary molar teeth using preformed metal (stainless steel) crowns. This technique has some advantages in the post-COVID-19 era because no local anaesthesia or tooth preparation is required. Topical fluorides, such as SDF, are effective at reducing caries. SDF is recommended by the ADA to treat advanced cavitated caries on any coronal surface of primary and permanent teeth. The use of SDF instead of other fluoride agents has grown in popularity in recent years [16]. In the present study, we found an increase in non-aerosol generating procedure-related queries. This finding can be explained by the growing popularity of these procedures.

Early childhood caries (ECC) is an aggressive form of tooth decay that affects children's primary teeth. ECC has a clear aetiology: high sugar intake, usually from a nursing bottle, combined with poor or non-existent oral hygiene, as a result of parents failing to brush or brushing insufficiently, results in an atypical pattern of caries attack, particularly on the smooth surfaces of upper anterior teeth in young children. Although professional guidelines recommend visiting a dentist at an early age, children are late to go for their first dental visit [25]. Our findings surprisingly show that search activity for ECC queries is actually decreasing. Similarly, we found a decrease in the RSVs for two other search queries, about root canals on baby teeth and mouthguards. Although people’s dental needs were not changed by COVID-19, there was an increase in searches for dental emergency queries during the pandemic. In addition, if patients have trouble accessing dental care providers, this may lead them to seek help for dental emergencies on the Internet. The statistically significant increase in the queries regarding dental emergencies, especially toothache and dental trauma, supports this.

## Limitation

These findings need to be interpreted with caution. The data pertain solely to Internet users' behaviour on the Google web platform and do not include queries from other search engine tools. Another issue was that it was not possible to access the raw data on GT; as a result, it is not possible to determine how many times a single person made paediatric dentistry-related queries. This could result in a duplication error in the records. Another issue is geographical and linguistic restrictions given there are many languages spoken across the world and the searches carried out on GT are worldwide. Therefore, studying queries only in English could be a limitation. However, the English language is one of the most prevalent languages worldwide. Other languages, such as Chinese, are also spoken by a large number of people but are more restricted geographically.

## Conclusion

People made many more dental emergency queries on the Internet during the pandemic. In addition, non-aerosol generating procedures such as the Hall technique are becoming increasingly popular with people.

## Data Availability

The datasets generated and/or analysed during the current study are available in the GT datasets. https://drive.google.com/drive/folders/1bEDba-c_8jLaSioy4lN5wIplsSA0P37H?usp=sharing.

## Abbreviations

ART: Atraumatic restorative technique
COVID-19: Coronavirus disease 2019
ECC: Early childhood caries
GT: Google Trends
WHO: World Health Organization
